# A Pt-Doped TiO_2_ Nanotube Arrays Sensor for Detecting SF_6_ Decomposition Products

**DOI:** 10.3390/s131114764

**Published:** 2013-10-30

**Authors:** Xiaoxing Zhang, Jing Tie, Jinbin Zhang

**Affiliations:** 1 State Key Laboratory of Power Transmission Equipment & System Security and New Technology, Chongqing University, Shapingba District, Chongqing 400044, China; E-Mails: cherrytie0123@hotmail.com (J.T.); Zhangjinbin023@126.com (J.Z.); 2 Chongqing Power Company, Beibei, Chongqing 400700, China

**Keywords:** gas sensor, Pt doped, TiO_2_ nanotube arrays, SF_6_ decomposition products

## Abstract

The detection of partial discharge and analysis of SF_6_ gas components in gas-insulated switchgear (GIS) is important for the diagnosis and operating state assessment of power equipment. The use of a Pt-doped TiO_2_ nanotube arrays sensor for detecting sulfur hexafluoride (SF_6_) decomposition products is proposed in this paper. The electrochemical pulse deposition method is employed to prepare the sensor array. The sensor's response to the main characteristic gaseous decomposition products of SF_6_ is evaluated. The gas sensing characteristic curves of the Pt-doped TiO_2_ nanotube sensor and intrinsic TiO_2_ nanotube arrays sensor are compared. The mechanism of the sensitive response is discussed. Test results showed that the Pt-doped nanoparticles not only change the gas sensing selectivity of the TiO_2_ nanotube arrays sensor with respect to the main characteristic SF_6_ decomposition products, but also reduce the operating temperature of the sensor.

## 1. Introduction

The excellent insulating and arc extinguishing properties of sulfur hexafluoride (SF_6_) gas greatly improve the dielectric strength when used as an insulating medium. SF_6_ has been widely utilized in gas-insulated switchgear (GIS) [1–4]. The reliability of GIS equipment is very high, however, its inevitable intrinsic defects continue to cause varying degrees of partial discharge (PD). Active gas produced by discharge accelerates insulation aging and corrosion of metal surfaces and may eventually lead to equipment failure. Many studies have shown that when a GIS insulation error occurs, the energy generated by discharge causes the SF_6_ gas to decompose and generate SF_4_, SF_3_, SF_2_, and various low-fluorine sulfides. These low-fluoride sulfides react with trace moisture and the oxygen present in the SF_6_ gas and generate SOF_4_, SOF_2_, SO_2_F_2_, SO_2_, HF, and other compounds [5,6]. The common methods used at present to detect and analyze SF_6_ partial discharge decomposition products include gas chromatography and infrared absorption spectrometry, but all these methods are offline laboratory detection methods, and on-site detection with these methods is difficult to implement.

Titanium dioxide nanotube arrays (TiO_2_NTs) are typical 3D nanomaterials that have numerous interesting physical and chemical properties. These materials are inexpensive and can thus be employed in numerous applications [7]. Studies have shown that compared with other nanostructure forms TiO_2_NTs have a large specific surface area and produce interesting nano-sized effects. TiO_2_NTs are utilized in photocatalysis, sensors, solar cells, *etc.* and exhibit a huge potential for development, having become one of the major topics in international nanomaterial research [8]. Miniature gas sensors prepared with TiO_2_NTs exhibit a fast response and high sensitivity. Several scholars have made great progress in this area in recent years, and TiO_2_NTs sensors are utilized to test for O_2_, NO_2_, H_2_, ethanol gas, *etc.* as sensitive materials [9–11].

A Pt-doped TiO_2_NTs gas sensor prepared through a pulsed electrochemical deposition method based on intrinsic TiO_2_NTs is developed in this study. The sensor's capability to sense the major decomposition products of SF_6_ is evaluated. Compared with the gas sensing properties of intrinsic TiO_2_NTs sensors, the Pt-doped nanoparticles change the gas sensing selectivity of the TiO_2_NTs sensor towards the main characteristic SF_6_ decomposition products.

## 2. Experimental Section

### 2.1. Preparation of Pt-Doped TiO_2_NTs

**Figure 1.**
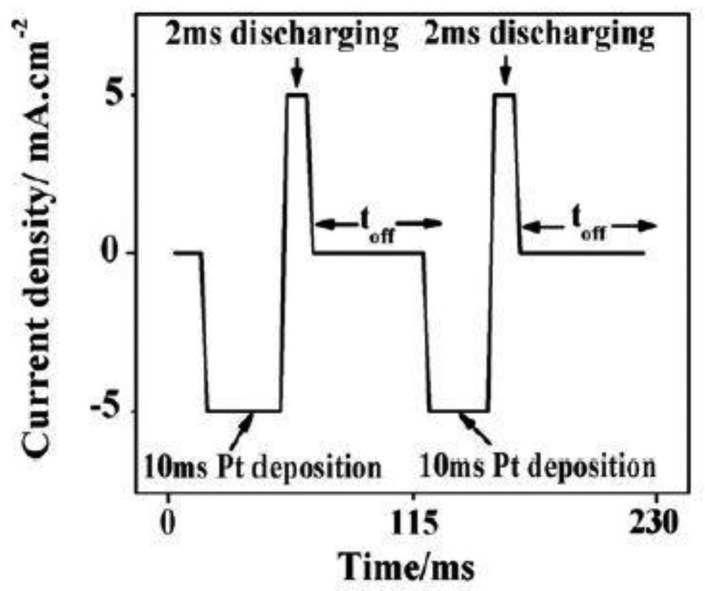
Current-time curve for the preparation of Pt-doped TiO_2_NTs.

The preparation of the Pt-doped TiO_2_NTs was based on electrochemical deposition on intrinsic TiO_2_NTs. The TiO_2_NTs were prepared by anodic oxidation [12]. With a conventional three-electrode system, Pt-doped nanoparticles were deposited onto TiO_2_NTs by pulsed electrodeposition. In the three-electrode system, the prepared intrinsic TiO_2_NTs is the working electrode (geometric area of 4.0 cm^2^). A Ag/AgCl electrode is the reference electrode, and a platinum electrode is the counter electrode. Electronic pulse signals were provided by an Autolab PGSAT32 instrument (manufacturer, city, state abbrev if US, country) in potential (constant) control mode. The current–time curve of the Pt-doped TiO_2_NTs preparation is shown in Figure 1. The electrolytes were prepared with pH = 4.4 aqueous H_2_PtCl_6_·6H_2_O (1 g/L) and H_3_BO_3_ (20 g/L) at 50 °C. The deposition time of the sample was set to 90 s to obtain moderately-sized Pt nanoparticles. Linear sweep voltammetry was employed at a current density of 5 mA/cm^2^ and continuous negative pulse for 10 ms. After each negative pulse, a short positive pulse (current density of 5 mA/cm^2^, continued for 2 ms) is discharged to the barrier layer capacitance. Then t_off_ = 100 ms time was used to restore the concentration of metal ions on the deposition surface.

### 2.2. Pt-Doped TiO_2_NTs Sensor Production

**Figure 2.**
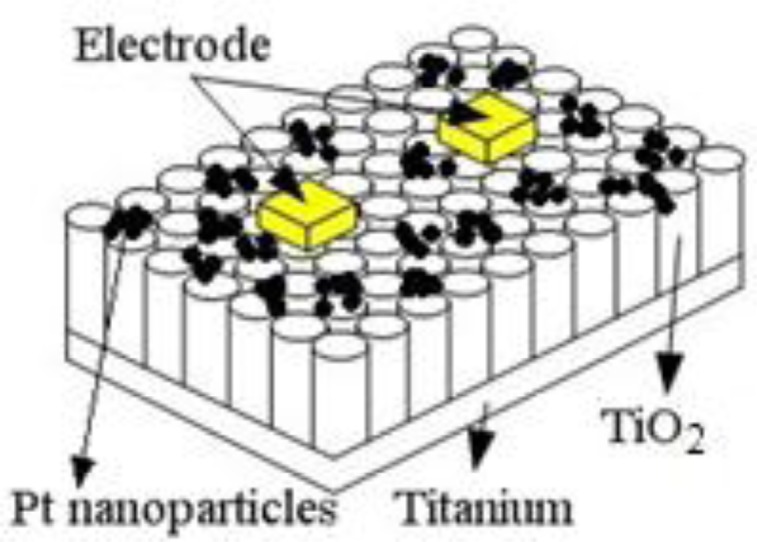
Sketch of the Pt-doped TiO_2_NTs sensor.

The TiO_2_NTs gas sensor is different from traditional gas sensors. TiO_2_NTs grow directly on the surface of a metal titanium plate and are not coated on a traditional Si substrate or an A1_2_O_3_ base. Therefore, high-temperature conductive silver glue was applied directly to the Pt-doped TiO_2_NTs surface to prepare the electrical contacts. The electrodes were closely pasted onto the TiO_2_NTs. Finally, the wires were connected to measure the surface resistance signal of the sensor. A sketch of the Pt-doped TiO_2_NTs sensor is shown in Figure 2.

### 2.3. Gas Sensing Test Device and Method for the TiO_2_NTs Sensor

**Figure 3.**
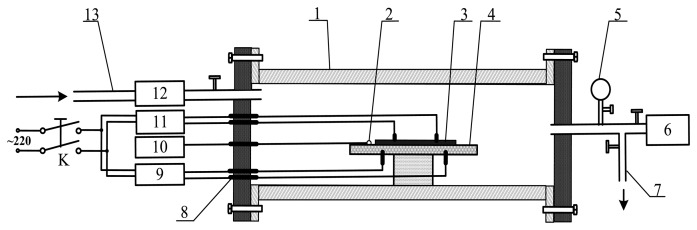
Detection test device utilized to measure the TiO_2_NTs sensor's response to the SF_6_ decomposition products. (**1**) Quartz glass tube; (**2**) thermal resistance probe; (**3**) carbon NT sensor; (**4**) ceramic heating slices; (**5**) vacuum form; (**6**) vacuum pump; (**7**) ventilation ducts; (**8**) terminals; (**9**) AC regulator; (**10**) temperature display apparatus; (**11**) impedance analyzer; (**12**) gas flow meter and (**13**) inlet ducts.

Figure 3 presents a schematic of the device utilized to measure the TiO_2_NTs sensor's response to the SF_6_ gas decomposition products. In the experiment, the calibration gases of the SF_6_ decomposition products were injected through the air intake. The gas flow meter controls and detects the flow rate of the measured gas, and the ceramic heater chip and thermal resistance probe control and measure the surface sensor temperature. The TiO_2_NTs sensor was then placed in a sealed quartz glass tube. The resistance characteristics of the sensor were determined with an impedance analyzer, and the resistance value of the entire process was recorded. The relative changes in the TiO_2_NTs sensor's resistance (*i.e.*, sensitivity) was calculated with the formula:

$$R\%=\overset{\left(R-R_{0}\right)}{\;}/\underset{R_{0}}{\;}\times 100\%$$

where *R* is the sensor resistance value after injecting the detected gas, and *R_0_* is the stable resistance in N_2_. The response time of the sensor is the time when the sensor's resistance reached 90% of the maximum value.

The adsorption of the TiO_2_NTs, air oxygen, and water vapor was considered. Dynamic measurements were performed in the experimental method to exclude the impact of these factors [11]. The concrete steps are as follows: prior to the gas sensing response experiment, high-purity N_2_ was injected at a flow rate of 0.1 L/min. The power supply of the heating sheet was turned on simultaneously. The regulator knob was adjusted to control the temperature of the sensor surface (to maintain the desired temperature) until the TiO_2_NTs sensor resistance value stabilized. The value was recorded as *R_0_*. Next, one of the gaseeous SF_6_ decomposition products, namely, SO_2_, was injected. The gas flow rate in the device should be consistent with the injection rate for N_2_. The sensor's resistance changed distinctly and achieved stability quickly (fluctuation near one resistance). The resistance value in this process was recorded as R. Lastly, high-purity N_2_ was injected at a flow rate of 0.1 L/min when the resistance of the sensor was stable; this procedure was performed until the resistance of the sensor gradually stabilized at a certain value. The value was recorded as *R_0_*.

## 3. Results and Discussion

### 3.1. Morphology of the Pt-Doped TiO_2_NTs Obtained through Characterization and Analysis

**Figure 4.**
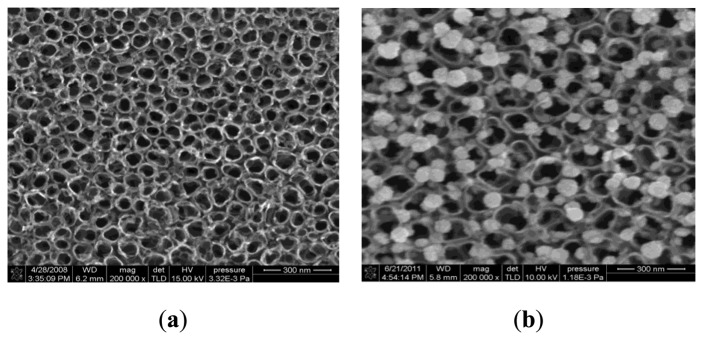
SEM images of the intrinsic and Pt-doped TiO_2_NTs. (**a**) Intrinsic TiO_2_NTs. (**b**) Pt-doped TiO_2_NTs.

A JEOL JSM-7000 field emission scanning electron microscope (SEM, JEOL, Japan) with an accelerating voltage of 10 kV was used to observe the sample. Figure 4 shows the SEM images of the intrinsic and Pt-doped TiO_2_NTs prepared by anodic oxidation and the proposed experimental method, respectively. Figure 4a shows the intrinsic TiO_2_NTs, and Figure 4b shows the Pt-doped TiO_2_NTs. The nanoparticles loaded between the tube and pipe in the Pt-doped TiO_2_NTs are Pt nanoparticles. The SEM images show that TiO_2_NTs prepared by the proposed experimental method are moderately sized and evenly distributed, thereby achieving the desired effect.

**Figure 5.**
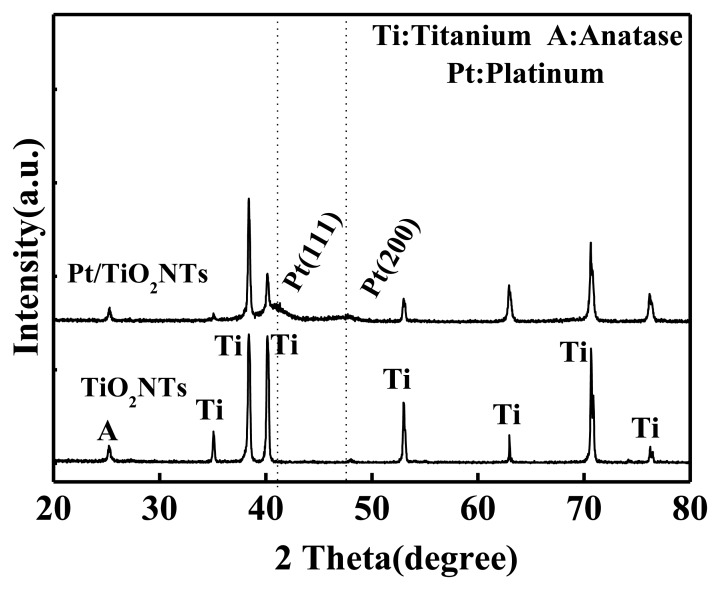
XRD of the Pt-doped TiO_2_NTs.

XRD was performed to analyze the sensor and to further validate whether the white nanoparticles in the SEM images are Pt [13]. The results are shown in Figure 5.

Figure 5 shows that a strong anatase (101) crystal plane peak exists at 2θ = 25.3° (A in the figure) in the intrinsic and Pt-doped TiO_2_NTs. In the XRD pattern of the Pt-doped TiO_2_NTs, a Pt (111) and a Pt (200) crystal surface peak exist at 2θ = 40.5° and 2θ = 46°, respectively. The XRD pattern of the intrinsic TiO_2_NTs did not have this peak. This result shows that pulsed electrodeposition is useful when Pt-doped nanoparticles are utilized with intrinsic TiO_2_NTs.

### 3.2. Influence of Operating Temperature on the Gas Sensitivity Characteristics of the Pt-Doped TiO_2_NTs Sensor

**Figure 6.**
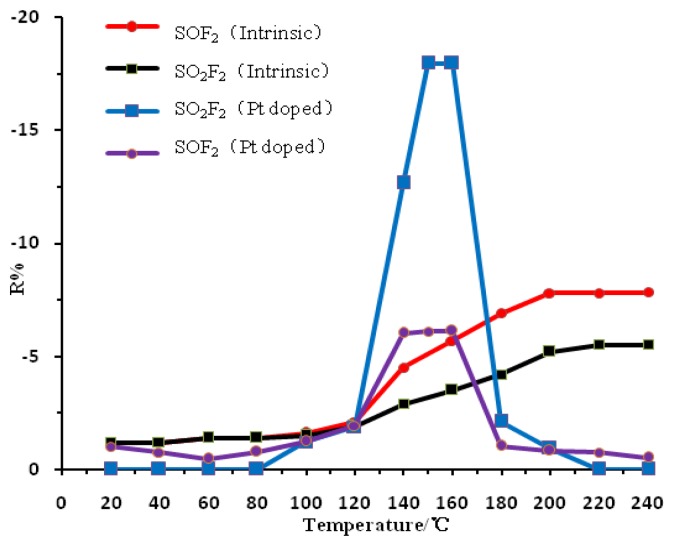
Sensitivity of the Pt-doped and intrinsic TiO_2_NTs sensors at different operating temperatures.

The performance of the metal oxide semiconductor gas-sensitive material is greatly influenced by the operating temperature. Metal or non-metal doping has a significant impact on the temperature characteristics of the metal oxide semiconductor material, therefore, examining the gas sensing response of Pt-doped TiO_2_NTs sensors to the SF_6_ partial discharge decomposition products (SOF_2_, SO_2_F_2_, SO_2_ gas) is necessary in determining the optimum operating temperature of the sensor. The prepared Pt-doped TiO_2_NTs sensor was placed in the test device described earlier (Figure 3). The surface of the sensor was then heated, and the temperature of the surface was controlled with the temperature control device. The gas sensing characteristics of the Pt-doped TiO_2_NTs sensor were tested in 50 ppm SOF_2_ and SO_2_F_2_ gas whose surface temperature changes from 20 °C to 240 °C. The result is shown in Figure 6.

Figure 6 shows the curve of the change in the resistance of the Pt-doped and intrinsic TiO_2_NTs gas sensors in response to the SF_6_ decomposition component gases SOF_2_ and SO_2_F_2_. The figure shows that the gas response value of the intrinsic TiO_2_NTs sensor to the SF6 decomposition product gases (*i.e.*, resistance change rate; R%) increases with the increase in the sensor surface temperature. The saturation temperature is approximately 200 °C, which is thus the intrinsic TiO_2_NTs sensor's optimum operating temperature. The R% of the Pt-doped TiO_2_NTs sensor also increases with the increase in the sensor surface temperature. The largest response value is generated when the temperature reaches 140 °C to 160 °C. As the temperature continuously increases the sensor response values begin to decline sharply. The response value becomes small when the temperature reaches 240 °C. This result indicates that the optimum operating temperature for the intrinsic TiO_2_NTs sensor is approximately 150 °C. This finding indicates that the Pt-doped nanoparticles not only reduces the optimal operating temperature of the sensor, but also changes the temperature characteristic curve significantly. Because the Pt doped sensor and SF_6_ decomposition products undergo redox reactions the sensor resistance decreases and the response values are negative. A detailed explanation can be found below in Section 3.4. When the intrinsic TiO_2_NTs sensor has reached the optimum operating temperature, the response remains unchanged even with a continuous increase in temperature, possibly because the gas sensor surface adsorption and desorption rates reach a dynamic equilibrium and thus, the sensor response remains unchanged.

The sensor surface microstructure and charge distribution is changed after the Pt nanoparticles are doped on it [14]. When the surface temperature of the TiO_2_NTs is higher than the optimum operating temperature, the Pt-doped nanoparticles improve the chemical desorption rate of the sensor surface. This phenomenon causes the desorption rate of the chemical adsorption of oxygen to be greater than its adsorption rate; the surface oxygen chemical adsorption density decreases, causing the response value of the sensor to decrease rapidly.

### 3.3. Response of the Gas-Sensitive Characteristics of the Pt-Doped TiO_2_NTs Sensor to SF_6_ Decomposition Component Gas

The sensor operating temperature was 150 °C. The gas-sensitive response curve of the Pt-doped TiO_2_NTs sensor was tested with SO_2_, SOF_2_, and SO_2_F_2_ gases at 30, 50, 70, and 100 ppm. The change rate in the sensor's resistance at various concentrations, *i.e.*, gas sensing response values, was calculated. The linear relationship between the change rate in the sensor's resistance and the gas concentration was investigated by the linear fitting based on the results. The concentration of the measured gas was also estimated by curve fitting based on the response value of the sensor.

**Figure 7.**
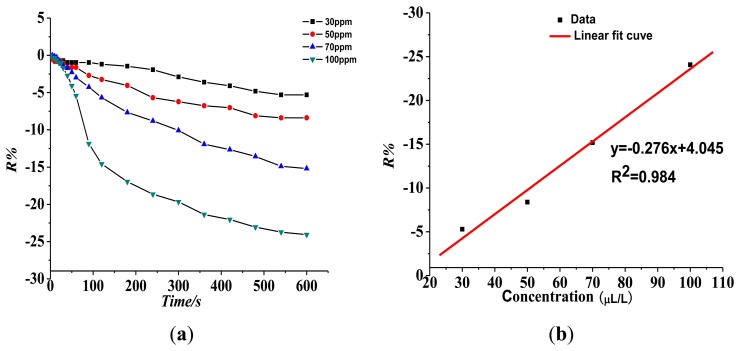
Response of the Pt-doped TiO_2_NTs sensor to different concentrations of SO_2_ at 150 °C operating temperature. (**a**) Response of the sensor's gas-sensing characteristics to different concentrations of SO_2_; (**b**) Linear relationship between sensor response and gas concentration.

**Figure 8.**
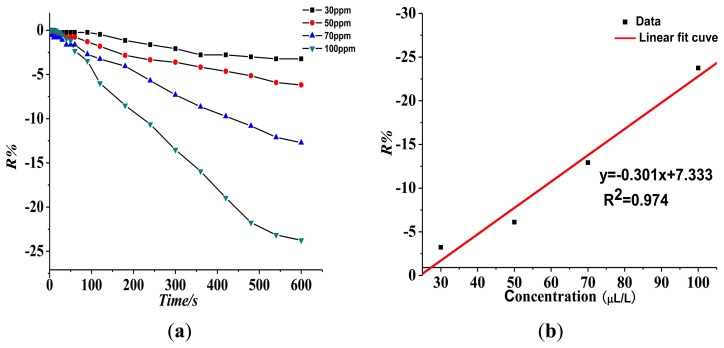
Response of the Pt-doped TiO_2_NTs sensor to different concentrations of SOF_2_ at 150 °C operating temperature. (**a**) Response of the sensor's gas-sensing characteristics to different concentrations of SOF_2_; (**b**) Linear relationship between sensor response and gas concentration.

**Figure 9.**
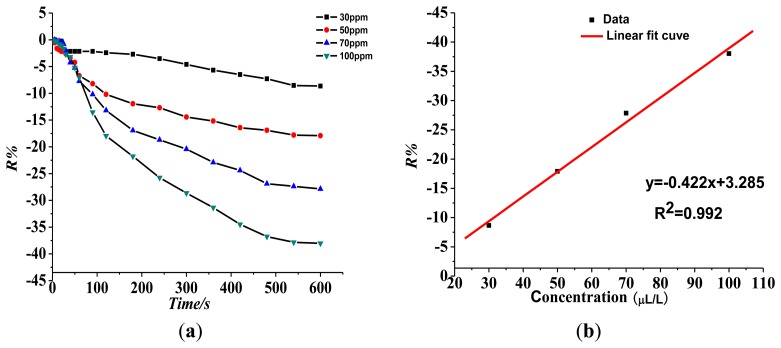
Response of the Pt-doped TiO_2_NTs sensor to different concentrations of SO_2_F_2_ at 150 °C operating temperature. (**a**) Response of the sensor's gas-sensing characteristics to different concentrations of SO_2_F_2_; (**b**) Linear relationship between sensor response and gas concentration.

(1) Response of the gas-sensing characteristics of the Pt-doped TiO_2_NTs gas sensor to SF_6_ partial discharge decomposition component SO_2_.
  Figure 7 shows that the resistance of the Pt-doped TiO_2_NTs sensor to 30, 50, 70, and 100 ppm of SO_2_ gas is 5.31%, 8.38%, 15.18%, and 24.07%, respectively. The linear fit function between R% and SO_2_ gas concentration is y = 0.276x + 4.045, and linear correlation coefficient R_2_ is 0.984.
(2) Response of the gas sensing characteristics of the Pt-doped TiO_2_NTs gas sensors to SF_6_ partial discharge decomposition component SOF_2_
  Figure 8 shows the Pt-doped TiO_2_NTs gas sensor's gas-sensing response curve to 30, 50, 70, and 100 ppm of SOF_2_ at 150 °C. Figure 8a shows that the resistance change rate with increasing concentration is 3.23%, 6.11%, 12.92%, and 23.75% at different tested concentrations of SOF_2_. The fitting curve is shown in Figure 8b. The linear fit function is y = 0.301x + 7.333, and linear correlation coefficient R_2_ is 0.974. This result shows that at a certain concentration range, a linear relationship exists between R% and SOF_2_ gas concentration.
(3) Response of the gas-sensing characteristics of the Pt-doped TiO_2_NTs gas sensor to SF_6_ partial discharge decomposition component SO_2_F_2_.
  Figure 9 shows that the resistance of the Pt-doped TiO_2_NTs sensor to 30, 50, 70, and 100 ppm of SO_2_F_2_ gas is 8.65%, 17.91%, 27.86%, and 38.02%, respectively. The fitting curve is shown in Figure 8b. The linear fit function is y = 0.422x + 3.285, and linear correlation coefficient R_2_ is 0.992.

### 3.4. Discussion on the Mechanism of the TiO_2_NTs Sensor's Gas Response Sensitivity

**Figure 10.**
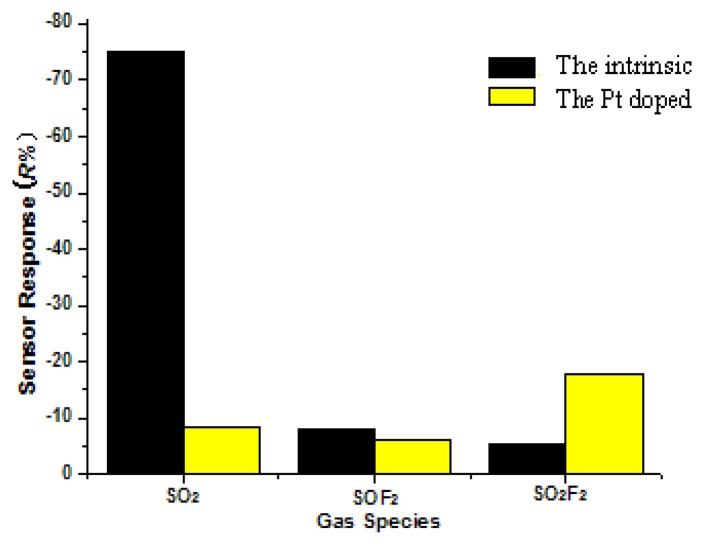
Response of the Pt-doped and intrinsic TiO_2_NTs to different SF_6_ decomposition components.

Figure 10 provides a comparison of the gas response of the intrinsic and Pt-doped TiO_2_NTs gas sensors to 50 ppm of the three SF6 decomposition productt gases (SO_2_, SOF_2_, SO_2_F_2_) at the optimal operating temperature. The gas response of the intrinsic TiO_2_NTs sensor to the three decomposition gases is discussed in Reference [12]. We find that the R% response value of the intrinsic and Pt-doped TiO_2_NTs to the three SF_6_ decomposition component gases is negative. This finding indicates that after the three gases were injected separately, the resistance of the intrinsic and Pt-doped TiO_2_NTs decreased, however, the three response values are different in size.

TiO_2_ is an N-type semiconductor and has many oxygen vacancies, thus, its gas sensitive effect is obvious and is generally considered a surface adsorption-controlled mechanism. Its response to the measured gas is caused by the chemisorption reaction between oxygen in the air and the TiO_2_NTs sensor surface. Oxygen ions exist in the grain boundaries between grains, thereby causing the grain boundary barrier to become higher, thus, the resistance of the TiO_2_NTs sensor increases, blocking the transfer of the carriers. When meeting the reducing gas or the electron supply gas, an oxidation reduction occurs between the surface adsorbed oxygen ions and the reducing gas. The number of adsorbed oxygen ions decreases sharply, the sensor surface potential barrier is reduced, carrier shifting is promoted, TiO_2_ resistance is reduced, and the gas sensing response is finally achieved.

Figure 10 shows that the R% response values of the intrinsic and Pt-doped TiO_2_NTs to the three SF_6_ decomposition component gases are negative. This result means that the resistance of the intrinsic and Pt-doped TiO_2_NTs decreased. The response mechanism shows that the three measured gases function as a reducing gas or an electron-donating gas. The reaction occurs as follows:

$$\text{R}+{{\text{O}}_{\text{ads}}}^{-}\Leftrightarrow \text{R}{\text{O}}_{\text{ads}}+{\text{e}}^{-}$$

where R is one of SF_6_ decomposition component gases, and O_ads_^−^ pertains to the oxygen ions adsorbed by the sensor surface.

The gas-sensing responses of the intrinsic TiO_2_NTs to the three tested gas are SO_2_ (74.6%) > SOF_2_ (7.82%) > SO_2_F_2_ (5.52%). This finding shows that the decreasing electron supply of SO_2_ gas is the strongest in the above-mentioned micro-oxidation reduction reaction (*i.e.*, the most likely to lose electrons), and the supply of SOF_2_ and SO_2_F_2_ gas is the weakest. The selectivity of the sensor is best for SO_2_ gas. The gas-sensing response of the Pt-doped TiO_2_NTs to the three tested gases is SO_2_F_2_ (17.91%) > SO_2_ (8.38%) > SOF_2_ (6.11%). The Pt-doped TiO_2_NTs sensor's response (*i.e.*, the selectivity of the sensor changes) to the three SF_6_ decomposition component gases (SO_2_, SOF_2_, SO_2_F_2_) changes significantly.

When the metal catalyst is doped, Pt functions as an oxygen storage point, such that it constantly provides Oads^−^ to the TiO_2_NTs sensor surface. Noble metal doping decreases the O_2_ + e^−^ → 2 O_ads_^−^ activation energy, thus, the optimum operating temperature point also decreases, and the reaction rate and gas sensitive effects are enhanced. As the catalyst particles on the surface of TiO_2_ sensitive body have a significant affinity interaction with the gas to be measured, the gas is attached firmly to the sensor's surface at a low temperature. The gas will “migrate” from the catalyst particles to the surface of the sensitive body and react with the adsorbed oxygen ions. Ultimately, the sensitivity of the gas sensor increases, and the sensor response rate is accelerated [15].

The three sulfide gases tested were SO_2_, SOF_2_, and SO_2_F_2_. Sulfides have a specific degree of toxicity to a noble metal catalyst, and the level of toxicity is related to sulfide valence and molecular structure. The toxicity of the three experimental gases from the strongest to the weakest is SO_2_ > SOF_2_ > SO_2_F_2_. S^6+^ within a certain range is non-toxic [16]. When the toxicity of SO_2_ passes through, the Pt-doped TiO_2_NTs sensor is poisoned. The specific process is as follows: First, SO_2_ is physical adsorbed on the active center of the catalyst; Second, a redox reaction occurs in the SO_2_ with the active ingredient; Third, the reaction produces the corresponding alkylene sulfide and sulfides, which block the active sites. The catalyst activity decreases in this complex series of processes [16]. The sensitivity of the almost non-toxic SO_2_F_2_ is greatly improved. The sensitivity of SOF_2_ is almost unchanged [12].

### 3.5. Recovery Test for the Pt-Doped TiO_2_NTs Sensor

**Figure 11.**
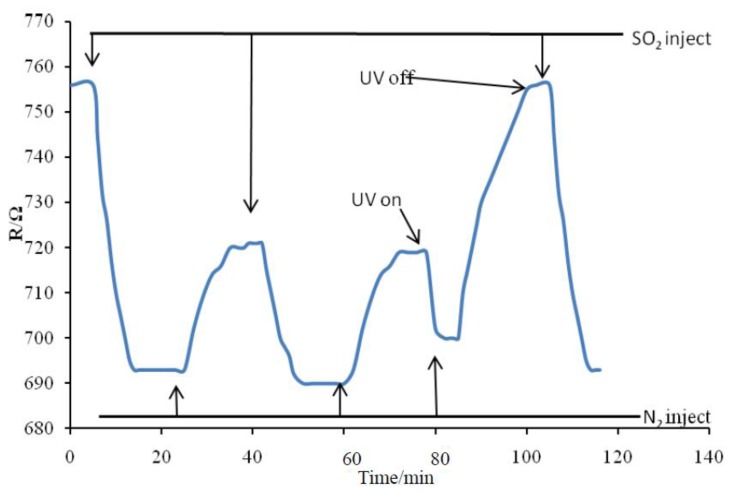
Recovery curve of the Pt-doped TiO_2_NTs sensor to SO_2_.

The recovery of the sensor in one of the SF_6_ decomposition product gases (SO_2_) at 150 °C was tested. The test results are shown in Figure 11. The sensor's resistance changed significantly when 50 ppm of SO_2_ gas was injected. When the sensor's resistance stabilized, pure N_2_ gas was injected. The sensor's resistance increased gradually, but did not revert to the initial resistance value. The above experimental procedure was repeated when the resistance value of the sensor stabilized again. The sensitivity of the sensor decreased greatly; the resistance value did not revert to the initial value, which indicates that the sensor underwent chemical poisoning. Afterward, irradiation with ultraviolet light was performed, and N_2_ gas was injected again. The resistance value of the sensor gradually increased and ultimately reverted to the initial value and became stable. When 50 ppm of SO_2_ gas was injected, the sensitivity of the sensor returned to its level in the first test. Although S^4+^ is toxic to the Pt-doped TiO_2_NTs sensor and may affect the initial resistance and sensitivity of the sensor, UV light irradiation resolves this problem by allowing sulfide ion desorption.

**Figure 12.**
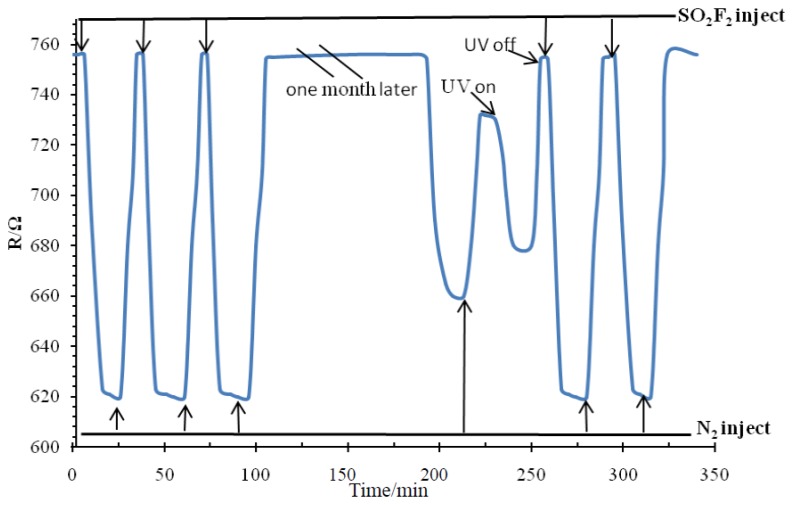
Recovery curve of the Pt-doped TiO_2_NTs sensor to SO_2_F_2_.

The recovery of the sensor in one of the SF_6_ decomposition product gases (SO_2_F_2_) at 150 °C was tested. The test results are shown in Figure 12.

This phenomenon is due to the residual thermal decomposition of SO_2_F_2_ molecules fixed in the TiO_2_ nanotube arrays as the result of chemical adsorption. The adsorption energy of chemical adsorption is much larger than the physical adsorption capacity. Therefore, ultraviolet light is used to desorb SO_2_F_2_ molecules attached on the sensor. The forbidden bandwidth of the ultraviolet photon energy is almost the same as that of many metal oxide semiconductors, hence ultraviolet radiation can be absorbed effectively by the TiO_2_NTs. The surface of the film and the inner portion undergo a range of physical and chemical processes. In the case of gas adsorption, ultraviolet radiation can be absorbed by the TiO_2_NTs through electron-hole pair excitation, thus increasing the carrier concentration and reducing the grain interface barrier. Through these processes, the TiO_2_NTs conductivity can be increased, and the resistor reduced. Ultraviolet radiation can be absorbed directly by gas molecules to produce desorption or stimulate chemical reactions between different types of molecules [17]. This method can improve sensor repeatability and reduce sensor chemical poisoning [12]. The recovery curve of SOF_2_ is very similar to that of SO_2_F_2_, so in the paper we do not repeat the corresponding discussion.

## 4. Conclusions

A Pt-doped TiO_2_NTs sensor was prepared by pulse electrodeposition; the sensor is based on an intrinsic TiO_2_NTs sensor prepared by anodic oxidation. The temperature characteristics of the Pt-doped TiO_2_NTs sensor were analyzed, and the gas sensing properties of three SF_6_ decomposition product gases were determined through experiments on their gas sensing properties. The gas sensing characteristics of the Pt-doped TiO_2_NTs sensor were compared with those of the intrinsic TiO_2_NTs sensor. The response mechanism was also discussed. The following conclusions were obtained:

(1) Under similar conditions, the Pt-doped TiO_2_NTs sensor's response to SO_2_F_2_ is strong, and its response to SOF_2_ and SO_2_ is weak. This result means that the Pt-doped TiO_2_NTs sensor exhibits good selectivity to SO_2_F_2_ gas.
(2) The temperature characteristic curve of the Pt-doped TiO_2_NTs sensor is different from that of the intrinsic TiO_2_NTs sensor. The optimum operating temperature of the Pt-doped TiO_2_NTs sensor is approximately 150 °C. When the temperature increases to 200 °C, the sensitivity response of the sensor declines sharply. When the temperature reaches 240 °C, the response value is very small (essentially zero).
(3) Comparative analysis of the gas sensing properties of the Pt-doped and intrinsic TiO_2_NTs sensors indicates that the Pt-doped nanoparticles change the gas sensing response of the intrinsic TiO_2_NTs sensor (*i.e.*, the selectivity of the sensor) to the three SF_6_ decomposition component gases. A preliminarily discussion of the response mechanism of the Pt-doped TiO_2_NTs sensor was presented.
